# Translating research into maternal health care policy: a qualitative case study of the use of evidence in policies for the treatment of eclampsia and pre-eclampsia in South Africa

**DOI:** 10.1186/1478-4505-6-12

**Published:** 2008-12-17

**Authors:** Karen Daniels, Simon Lewin, The Practice Policy Group

**Affiliations:** 1 Health Systems Research Unit, Medical Research Council, Durban, South Africa; 2 Department of Public Health and Policy, London School of Hygiene and Tropical Medicine, London, UK

## Abstract

**Background:**

Few empirical studies of research utilisation have been conducted in low and middle income countries. This paper explores how research information, in particular findings from randomised controlled trials and systematic reviews, informed policy making and clinical guideline development for the use of magnesium sulphate in the treatment of eclampsia and pre-eclampsia in South Africa.

**Methods:**

A qualitative case-study approach was used to examine the policy process. This included a literature review, a policy document review, a timeline of key events and the collection and analysis of 15 interviews with policy makers and academic clinicians involved in these policy processes and sampled using a purposive approach. The data was analysed thematically and explored theoretically through the literature on agenda setting and the policy making process.

**Results:**

Prior to 1994 there was no national maternal care policy in South Africa. Consequently each tertiary level institution developed its own care guidelines and these recommended a range of approaches to the management of pre-eclampsia and eclampsia. The subsequent emergence of new national policies for maternal care, including for the treatment of pre-eclampsia and eclampsia, was informed by evidence from randomised controlled trials and systematic reviews. This outcome was influenced by a number of factors. The change to a democratic government in the mid 1990s, and the health reforms that followed, created opportunities for maternal health care policy development. The new government was open to academic involvement in policy making and recruited academics from local networks into key policy making positions in the National Department of Health. The local academic obstetric network, which placed high value on evidence-based practice, brought these values into the policy process and was also linked strongly to international evidence based medicine networks. Within this context of openness to policy development, local researchers acted as policy entrepreneurs, bringing attention to priority health issues, and to the use of research evidence in addressing these. This resulted in the new national maternity care guidelines being informed by evidence from randomised controlled trials and recommending explicitly the use of magnesium sulphate for the management of eclampsia.

**Conclusion:**

Networks of researchers were important not only in using research information to shape policy but also in placing issues on the policy agenda. A policy context which created a window of opportunity for new research-informed policy development was also crucial.

## Background

### Research utilisation in policy making

The importance of basing health care decision making at both a clinical and a policy level on the outcome of sound research studies, rather than only on clinical experience and pathophysiological understanding, is increasingly being recognised [1,2]. Davies & Nutley [3] suggest that this shift in approach to decision making has been encouraged by the growth of evidence based medicine. Proponents of this approach suggest that decisions at a policy level about resource allocation ought to be made on the basis of "what works"[4-6]. In turn they believe that "what works" can be determined on the basis of sound research evidence from the evaluation of health care interventions, particularly that based on systematic reviews of randomised controlled trials (RCTs) [1,2]. It is argued that decisions made on the basis of such research evidence can be not only cost saving [5], but also life saving [1].

Although the view that policies should be informed by research is widespread, and the pool of evidence on which to base decisions is growing, [1,2], none of these positive factors have lead to the automatic uptake of research into policy making [7]. The literature recognises that the relationship between knowledge production (research that produces evidence) and knowledge utilisation (evidence used in policy making, programme implementation, programme design, etc.) is complex [6,8] with many impediments to the use of research in policy [9-11]. The process of research utilisation in health care policy making has therefore, in it itself, become an area of study [9,12] in an attempt to find ways of increasing the uptake of research findings.

Much of what has been written on the use of health care research by policy makers and managers takes the form of theory and opinion. However some empirical research in this area has been conducted. Two recent systematic reviews have synthesised findings from qualitative studies of evidence use [9,12]. The first [9] focused on studies with health policy makers while the second included both health policy makers and health care managers [12]. A summary of the findings from these reviews is presented in Table 1.

**Table 1:** Factors identified as influencing research use: a comparison between the findings of this study and that of two earlier systematic reviews.*

***Systematic review 1 ***[9]	***Systematic review 2 ***[12]	***This study***
Interaction (or the lack of interaction) and trust between policy makers and researchers	Interaction between policy makers and researchers Trust in the research and the researcher	An interactive relationship between policy makers and researchers in which the researchers are able to receive the evidence and interpret it for members of the bureaucracy

The timeliness, relevance and quality of the research	The quality, timing and timeliness of the research; and the perceived relevance of the research	The evidence being regarded as being of good quality and therefore trustworthy Appropriate evidence available at the right time, in this case when a solution to the problem of the high maternal mortality rate was being sought

The inclusion of effectiveness data	-	-

The political environment including political (in)stability and community pressure	-	A political environment that is conducive to policy making

The extent to which research confirmed existing policies	-	-

Bureaucratic processes including power and budget struggles	Political and bureaucratic conflict	A bureaucracy that is open to change rather than obstructive

The availability of research summaries with clear recommendations	Publishing findings in a manner that is accessible beyond a scientific audience	-

-	The importance of management support	-

-	The skill and attitude of those receiving the research	-

-	The existence of policy networks	A functioning policy network that includes researchers, policy makers and bureaucrats

-	-	The evidence being received in the context of a positive attitude towards research utilisation, particularly with regard to evidence from randomised controlled trials and systematic reviews.

* For each review, and for this study, we have attempted to list each of the factors identified as influencing research uptake into policy making. Where similar factors were identified by each review/study, these are listed in the same row of the table. An empty cell indicates that the relevant review/study did not identify this factor as important.

While these reviews present the best evidence we have on research use, they are limited by the empirical studies available. Lavis et al [12] conclude that there is a paucity of sound research in this field, arguing that the yield of research is neither plentiful, rigorous (more than one data collection method) nor consistent (in the factors identified across studies as influencing evidence use). This paucity of rigorous studies is particularly striking for low and middle income countries (LMICs): Lavis et al [12] identified only one study from these settings out of seventeen included in the review while Innvaer et al [9] found only four LMIC studies from a total of twenty four. This is despite the issue of research utilisation being particularly pertinent in LMICs which face a scarcity of resources coupled with a high disease burden. While research has identified effective and affordable interventions for many of the key health problems in these countries, these are often not implemented or are discarded in favour of unproven interventions [13-16]. Maternal health in South Africa is no exception to this. Numerous reports and papers have, on the one hand, highlighted effective maternal health interventions while, on the other, noted failures to implement these programmes in many settings [17-20].

### The maternal health policy context in South Africa

South Africa's health system has been dramatically and negatively impacted upon by its apartheid past [21]. Maternal health services, and other health services, have suffered as a consequence [22]. Prior to the change of government in 1994, there was no single national maternal health policy, let alone coordinated clinical protocols. Instead, each institution decided upon its own protocols. This situation changed with the transition to democracy when the health of women and children was recognised as a priority by the new government [22]. In the process of transition, concerted attempts were made to transform the health system overall and in particular to affect policy reform [23].

### The problem of eclampsia and pre-eclampsia in South Africa

Pre-eclampsia and eclampsia (see below) are important causes of maternal and infant morbidity and mortality globally. Over 63 000 women are estimated to die annually after eclamptic convulsions, with most of these deaths occurring in low and middle income countries [24-26]. Hypertension in pregnancy also remains a leading cause of maternal death in South Africa. In 1998 hypertension in pregnancy was reported as the highest primary obstetric cause of maternal death (23.2% n = 131), with eclampsia accounting for the highest proportion (59% n = 58) of deaths within this group [17]. Subsequent enquiries into maternal deaths reported similar findings [18,27]. Hypertension related deaths were surpassed only by non-pregnancy related infections (31.4%), amongst which death from AIDS-related illness is included [17]. Hence hypertension remains the leading direct primary obstetric cause of maternal mortality [28].

Pre-eclampsia has been defined as a disorder of pregnancy that involves multiple systems within the body. It is usually associated with raised blood pressure, or hypertension, and protein in the urine (proteinuria). The exact cause of pre-eclampsia is not well understood. When severe, the condition can involve the woman's liver, kidneys, clotting system or brain. The placenta, that provides nutrients to the baby, is also often involved, with an increased risk of poor growth and early delivery for the baby. Eclampsia – a potentially life threatening condition – is the occurrence of a convulsion or fit during pregnancy in association with pre-eclampsia. It may occur if pre-eclampsia is not controlled [29].

### Effective treatment for eclampsia and pre-eclampsia: The evidence

Research evidence offers a treatment solution to the high maternal mortality rate of eclampsia. Two large multi-centre RCTs [19,30,31] and three systematic reviews [32,29,33] have shown the effectiveness of magnesium sulphate in treating eclampsia and pre-eclampsia. Concerns have been expressed internationally, however, that this evidence is not necessarily translated into policy and/or practice [15,34,35]. Using the example of magnesium sulphate, Garner et al [5] argue that while in 1995 [30] a large trial showed that this drug was the most effective treatment for eclampsia, other less effective therapies were still being used in obstetric practices in one third of the world.

### Aim

This paper reports on a sub-study within a three country study of research utilisation[36]. The paper explores the actual and perceived utilisation of research information, in particular findings from RCTs, in policy making and clinical guideline development for the treatment of eclampsia and pre-eclampsia in South Africa over the period of 1970 to 2005. Treatment policy for these conditions was selected as an example of maternal health care policy development in which an effective, evidence-based intervention was available that was, in some respects, easier to implement than other obstetric practice changes, such as the active management of the third stage of labour. Drawing on theoretical approaches to research utilisation and policy making, the paper aims to contribute to an empirical understanding of research utilisation in the process of policy making in LMICs. There are several reasons why the South African setting provided an ideal opportunity to study a contemporary relationship between policy making and research in a developing country. Firstly, eclampsia and pre-eclampsia had been identified as a national problem. Secondly, RCTs and systematic reviews had recently provided robust evidence of an effective treatment. Thirdly, national maternal health policy had recently been developed.

### Theoretical and analytical approaches

The literature on research utilisation suggests an inherent complexity in the process by which research is translated (or not) into policy and management decisions [7,8]. For example specific research may be turned to because of a need to solve a particular problem, or policies may gradually be more reflective of research as policy makers become influenced by the finding over time [37]. Within the policy making process, policy makers adopt, adapt and act on research [38]. They may use research evidence instrumentally (directly), conceptually (indirectly) or symbolically (as justification) [39]. Research can also be used at different stages of the policy making process such as prioritisation, development or implementation [40].

It has also been argued that research utilisation is influenced by many contextual factors such as the social and political environment in which policy making is taking place [10,16,38,41]. Yet as Bowen and Zwi [38] suggest, researchers may not take account of the broader context. They argue that:

"The social and political context and the many forces at work in the policy environment provide challenges to integrating evidence into policy and practice. Researchers often do not see or recognize these factors" [38](p. 0601).

Since research is only one factor influencing policy making, we needed to explore the process of policy making as a whole in order to understand the place of research. We also wished to move beyond a simple description of where and how research is utilised in policy making. To do so we turned to the literature on the policy making process. Two models, that of agenda setting in policy making [42] and influences on the policy making process [40] were used as lenses through which to understand our data. We briefly describe these models below.

We firstly drew on the analytical framework developed by Kingdon [42] and applied widely within the health domain [23,43,44]. This framework was not developed for the analysis of research utilisation specifically, but for understanding how issues enter the policy agenda. Kingdon [42] suggests that three 'streams' shape agenda setting within governments and other institutions – the problem stream, the policy stream and the stream of politics. These streams, he argues, are largely unrelated to each other. In the problem stream are those issues defined or perceived by policy makers and other officials as priority problems. In the politics stream are various stakeholders and interest groups who put forward particular points of view. Finally, the policy stream selects from problems and politics those issues that will be shaped into policies, based on factors such as acceptability and feasibility. However, these policies may not relate immediately to what are seen as key problems within the problem stream. Policy change can occur when the three streams merge, sometimes as a result of the activities of advocates or policy entrepreneurs. This Kingdon [42] calls a 'policy window', when either the problem is so large or there is a change in the political stream and policy entrepreneurs have the chance to advance their proposals onto the policy agenda. We drew on Kingdon's [42] framework to explore how maternal health entered the policy agenda, leading to policy and guideline formulation.

We also drew on a political science framework that distinguishes "three general categories of influence on the policymaking process" [40](p. 141). These categories of influence include ideas – values, research etc.; interests – actors with a vested interest in the policy and those who will benefit from or be harmed by the policy; and institutions – the policy legacies and characteristics of the policy. In addition this framework highlights the importance of events- occurrences such as a change of government or a public health emergency that may influence opinions and attitudes and may also open and close opportunities for change. Within this framework, research is only one component contributing to the ideas that influence policy making. The framework provides a useful lens through which to retrospectively assess the weight of the influence of research in policy making at a particular moment in time, as compared with all of the other influences or factors operating at the same time. We utilised this framework to understand the range of factors influencing policy making for maternal health.

## Methods

This study adopted a qualitative approach to understand the phenomena under investigation as experienced by the actors involved in it [45]. Exploring actors' own accounts of their experiences enhances understanding of the processes, rather than simply the outcomes, of research utilisation. Given the complexity of the issues involved, a case-study methodology was used to explore the topic [46]. Throughout we attempted to adopt a reflexive stance by maintaining an awareness of our influence over the research process and outcomes [47].

### Data collection

The case study is built around a triangulation [48] of three data collection methods: a review of the recent policies, the development of a timeline and fifteen key informant interviews with local researchers and policy makers. All of these methods have been used in previous studies of the utilisation of research information in health policy [7].

#### Policy Review

Copies of all contemporary national policies and guidelines were obtained from the National Department of Health. These were reviewed with the aim of establishing the extent to which research information had been implicitly and explicitly used [40]. This was done through checking the references of each policy and the extent to which the use of research information was mentioned. Whether or not the policy recommended magnesium sulphate for the treatment of eclampsia and pre-eclampsia was also noted.

#### Timeline

A timeline of key events in the management of eclampsia and pre-eclampsia in South Africa was constructed iteratively. During each interview respondents were asked to comment on or add to the timeline. It was also shared with colleagues and any further information was incorporated. Relevant bibliographical and conference databases, as well as the websites of organisations such as the National Department of Health in South Africa and the Cochrane Collaboration, provided additional information.

#### Interviews

Between 2004 and 2005 individual qualitative interviews were conducted by KD and SL with fifteen local researchers and government officials (past and present). These interviews were audio recorded and then transcribed verbatim. Neither of the interviewers were known among respondents as an advocate of evidence-based medicine. The interviews were structured around a flexible interview schedule designed collectively by the broader research team to address all facets of the research question. Each interview broadly covered the following topics: the respondent's background; their knowledge of national policies; their knowledge of and involvement in the policy development process; their understanding of the various influences on the policy process and content including stakeholder involvement, prevailing values and research information. The respondents were purposively sampled on the basis of being influential and/or knowledgeable in the process of policy making and guideline development for maternal health, and included both obstetricians and midwives. They were identified through colleagues working in the field and using a 'snowballing' approach [49]. Within this process attention was given to fair dealing and seeking out negative cases [47]. During the course of our data collection it became clear that we were interviewing people who shared similar opinions regarding the use of research evidence in the maternal health policy process. In fact one respondent referred to there being a "club" within the obstetric community. We therefore actively sought respondents who might have held differing views. However we only identified one such individual.

Our respondents had all been trained in either midwifery (4) or obstetrics and gynaecology (11). They were all either active in the policy and guideline development process or were very knowledgeable about this area. Four respondents were currently or formally employed by the National Department of Health. One respondent was a practising midwife and nursing tutor. The other eight were academic researchers (professors and associate professors). One respondent had held a senior position both in the national department and as an academic researcher and was able therefore to reflect on the research question from both perspectives.

In reporting on our findings below, we use the abbreviation 'DoH' to indicate data extracts from interviews with current or former staff of the National Department of Health. The abbreviation 'Acad' indicates extracts from academic researchers. The number after the abbreviation indicates the respondent number in each group.

### Analysis

A thematic content analysis of the verbatim transcripts was conducted [50]. The process was led by KD, supervised by SL and internally validated by the wider three country research team. After immersion in the transcripts, these were coded for both latent and manifest themes [51]. Data extracts assigned to each code were cut and pasted into a Word document, which was shared and checked between KD and SL. Out of this process a narrative account of the data was written up as the key research findings, some of which are presented here. The findings are illustrated further by data extracts selected on the basis of being representative and/or interesting illustrations of each of the themes [52]. Although our analysis was primarily inductive, we drew on several analytic frameworks from the policy literature to better understand how research evidence informed policy-making in this context, as described above.

### Ethical issues

The main study [36] received approval from ethics committees at the Medical Research Council of South Africa, the London School of Hygiene and Tropical Medicine, the University of Zimbabwe, Eduardo Mondlane University and the World Health Organisation. This sub-study also received approval from the ethics committee chairperson at the University of Cape Town.

All participants in the study signed a consent form after being given a written study information sheet and a verbal explanation of the consenting process.

## Findings

### Research utilisation in contemporary maternal health policies and guidelines: a document review

Since 1994 the following relevant guidelines for maternal care, including the management of eclampsia and pre-eclampsia, have been produced:

• *Guidelines for Maternity Care in South Africa: A manual for clinics, community health centres and district hospitals*, (2000 & 2002)[53]

• *Saving Mothers: Policy and Management Guidelines for Common Causes of Maternal Deaths*, (2001) [54]

• *Saving Mothers: Second Report on Confidential Enquiries into Maternal Deaths in South Africa, 1999–2001*, (2002)[18]

The first set of guidelines (Maternity Care), were generalised and aimed at primary care practioners. In contrast the second set (Saving Mothers) was specific to the treatment of priority maternal health problems as identified by the Confidential Enquiries. Furthermore, the "Saving Mothers" guidelines were aimed at all levels of care including tertiary care.

These documents are very different in the manner in which they reflect their use of research evidence. Both editions of the "Guidelines for Maternity Care" include in their introduction a statement reading:

"The guidelines are based on the best available evidence from published research, modified where necessary to suit local conditions. References are not given, but are available from the authors on request. Specifics of management and drug dosing are not cast in stone, and can be modified according to the experience of the reader and new evidence". (p. 8 in both editions)

They also both refer to the Cochrane Library as a site for further reading. In other words, although the guidelines claim to be based on evidence, it is left to the person reading the guidelines to verify if they so wish. The two documents suggest the use of evidence, but the nature of this use is not made explicit. In the guidelines themselves, though, magnesium sulphate is the only drug recommended for the treatment of severe eclampsia, imminent eclampsia and eclampsia. Guidelines for its administration are provided.

The two "Saving Mothers" documents are far more explicit in their use of research evidence. The "Policy and Management Guidelines" (2001) notes that it is "customary to grade the evidence, on which clinical statements are based, according to the strength of such evidence" (p. 5). The document then goes on to explain the grading levels, e.g. "**Grade A **Evidence from randomised controlled trials". Within each guideline section full references to the study from which each specific recommendation is drawn, and the grade of the evidence is given, for example:

"Magnesium sulphate is the best drug to arrest and prevent further convulsions Grade A evidence. (The Eclampsia Collaborative Group. Lancet 1995; 345:1455–63)" (p. 28)

The policy document review therefore showed very concrete and explicit examples of research utilisation in policy making and guideline development. It also shows the strengthening of the use of research evidence in policy documents over time. The interview and historical data presented below explain the process through which these policies came about.

### Policy making for maternal care in South Africa

#### • Filling a policy vacuum and standardising national care

There was no national policy or management guideline for maternal care until the publication of the 'Guidelines for Maternity Care in South Africa' in 2000, followed by the 'Saving Mothers Policy and Management Guidelines for Common Causes of Maternal Deaths' in 2001. Prior to this, as respondents explained, the obstetric departments of the various medical universities around the country tended to draw up their own guidelines and protocols to be used at hospitals and maternity services attached to these institutions. As one respondent argued, these institutions tended to pursue "their own identities even in terms of protocol" (DoH 2). Both nursing and medical students were taught practices that followed the protocol of the training institution in which they were enrolled. Consequently, there was no standardisation of practice across the country, causing difficulties when health professionals from different practice backgrounds had to work together in one setting:

"So this [national policy] then starts saying, 'This is what is accepted as practice in South Africa'. So that even if you come from [the University of] Cape Town and you decide you're going to give 20 drops, and another one comes from Medunsa [the Medical University of South Africa] and say we give 30 drops, then [the policy] will say: 'These are the drops that you must give'. So that the nurse, who is working with the doctor, knows that this is what has to be done. Not to be shouted at because she's running with 25 drops and at Medunsa they say 'not giving enough', [while] Cape Town says this: 'You're giving more than enough'... " (DoH 2)

This lack of national standardisation was compounded in some settings by foreign doctors using yet further protocols developed in their own countries.

The structure of the South African health system means that medical specialists are often situated only in urban tertiary hospitals, thus not reaching all women in need of specialist care. Our respondents indicated that one intention of the guidelines was to expand care by allowing for midwives and doctors who had not received specialist training, and specialists who had not trained within the country, to all be able to deliver care of a similar quality to that that might be provided by a specialist (DoH 1). Furthermore, they suggested that the guidelines were also intended, through the involvement of academics in their writing and reviewing, to influence the training of health care providers across the country. We discuss this in more detail below.

#### • A change in direction for national maternal health care

Our interview data suggested that the change in government in South Africa in 1994 was a crucial watershed for policy development. As shown in the timeline (Table 2), the national policies and guidelines for maternal care were published six and seven years after the first democratic election. Policy development was described by respondents as an aspect of democratic reform, with policies being formulated throughout health care, not just for maternal care, as has also been noted elsewhere [23]. One respondent (Acad 7) suggested that the change in government ushered in a new openness to discussions between researchers and government regarding maternal health and other policies. New staff employed by the National Department of Health were seen by several respondents as having different values to those who had been there before. For example, one respondent suggested that record keeping under the previous government was designed to hide the health problems of the poor, but that the new cohort of people employed in the department had experienced these problems personally and wanted to correct them. These respondents, who had been employed in the National Department during this phase, spoke of wanting to improve health for all:

**Table 2:** An abbreviated timeline of key events

**Date**	**Key Events in** **The evolution of policy and guidelines for the treatment of eclampsia and pre-eclampsia in South Africa**
1955	First Pritchard case series published, showing effectiveness of MgSO4. Updated every 10 years until 1984
1968	British physicians in Hong Kong suggest use of diazepam
1970's	MgSO4 introduced into obstetric care at key medical faculties in South Africa
1979	Obstetrics criticised by Archie Cochrane as being least evidence based medical speciality
1980's	Use of MgSO4 spreads through teaching and inter-institutional contact between academics. International divisions on the choice of anti-convulsant are reflected in the country
1980's-90's	South African researchers become increasingly connected to the international obstetric research community and simultaneously the local research output increases Although provinces and institutions have their own policies there are no national policies or guidelines
By early 1990's	MgSO4 in widespread use in SA for treating eclampsia
1990	Randomised controlled trial, of MgSO4 vs phenytoin for eclampsia conducted in South Africa published in British Journal of Obstetrics and Gynaecology 1990 Feb; 97 (2): 104–9.
1992	Pregnancy and Childbirth Group, first Cochrane Review Group to be registered
1993–95	South African researchers collaborate in Eclampsia Trial at local trial sites
1994	Change of government promotes new focus on maternal health and openness to academic involvement in policy making
**1995**	**Collaborative Eclampsia Trial published**
1995	Senior obstetricians in South Africa publish editorial on implications of Collaborative Eclampsia Trial
1996	Maternal health organised into a separate directorate within national DoH Academic advocacy for maternal mortality monitoring followed by the appointment of the first National Committee for Confidential Enquiry into Maternal Deaths (NCCEMD)
1999	First NCCEMD report published. Eclampsia accounts for highest percentage of the deaths due to hypertensive disorders of pregnancy, which is the second largest cause of maternal deaths. National policy and guidelines recommended.
*2000*	*First national "Guidelines Maternity Care" published. Evidence is referred to but not referenced*
*2001*	*NCCEMD publishes policy and management guidelines for common causes of maternal deaths. MgSO4 recommended and use of evidence made explicit*. South African researchers collaborate in Magpie Trial at local sites. South Africa is the regional trial co-ordinating centre.
**2002**	**Magpie Trial published**
2003	Following on from trial, MgSO4 is recommended for women with moderate to severe pre-eclampsia, where it can be administered safely, in South African Medical Journal.

"So there was a need to then say it was one country, and therefore, we need to reform this [country] and we need to have policies that respect everyone irrespective of race, colour or creed. So I think that is the one aspect. I think also the fact that many of the people who then came into government, came from underprivileged communities. So it was important and necessary to try and change the conditions of those communities." (DoH 2)

#### • Influence and values of a small academic obstetric network

Following the shift in values regarding the delivery of health care, professional networks in South Africa, including the South African Society of Obstetrics and Gynaecology, the College of Medicine and the Priorities in Peri-Natal Care Conferences, were influential in placing maternal health on the policy agenda. The new cohort of staff, appointed into key positions within the Directorate of Maternal and Child Health within the National Department of Health, had long standing links with these networks. In addition, respondents described how a small group of researchers involved closely in these networks were able to convince the National Department, and in particular the incoming Minister of Health (Dlamini-Zuma), of the need to make maternal death notifiable so as to allow the extent of the problem and its causes to be explored systematically. This group lobbied for the assessment of the leading causes of mortality through a confidential enquiry into maternal death. Thus in 1996 the first National Committee for the Confidential Enquiry into Maternal Death was appointed, chaired by Prof. Jack Moodley, a leading figure within national obstetric networks. Respondents described the outcome of this enquiry as particularly significant for policy development with regard to the management of eclampsia. Firstly, the first Confidential Enquiry found that hypertension was the leading cause of maternal mortality (prior to the escalation in AIDS related deaths). Secondly, it recommended that a national policy be written. Following from this, the National Department began drafting the 'Guidelines for Maternity Care in South Africa' in 1998. At the launch of the first report of the Confidential Enquiry, members of the College of Medicine volunteered to write clinical management guidelines for the treatment of the ten most common causes of maternal death. Thus two national policies were published within a year of each other.

The importance of this obstetric network, though, was not only their influence in bringing attention to the need for maternal care policies and their willingness to assist in developing these, but also the values that they brought to this development process. Beyond regular local contact, key members of this group were also tied into international networks for the promotion of evidence based medicine, such as the Cochrane Collaboration and the National Perinatal Epidemiology Unit in Oxford. One respondent described the close links between the South African researchers and these international evidence-based practice networks:

"We actually invited Murray Enken, who was attached to the Oxford Database [of Perinatal Trials]. And subsequent to that Iain Chalmers, who's the editor, was invited to attend [the annual Priorities in Perinatal Care conference]. So we were sort of, I think from the word go, when the Oxford Database became available for use, we were part of it, we were aware of it, we were using it, and I think quite a few South Africans became involved on their editorial board and as editors or reviewers, or whatever." (Acad 1)

Within this network of South Africa researchers, the concept of evidence based medicine had been diffused extensively:

"No, it [the clinical maternity care guidelines] was purely evidence based. We really tried to be as scientific as possible... the Oxford database was used very extensively, and subsequent to that the Cochrane database. So we tried to stick as close as possible to evidence-based medicine and not sort of traditional ways and means of dealing with things, but really to make it, have it scientifically founded." (Acad 1)

"At that stage, I was a member of the society [of obstetricians and gynaecologists] and attended all their congresses, and I was director of [a] ...research unit... So of course, you wanted to do the best practice. You wanted to use the best protocols. You wanted to do evidence based medicine." (Acad 6)

The view that policy making should be based on best available research evidence was strongly held. Best evidence was understood to be that which was derived from randomised controlled trials and systematic reviews:

"And if you look at the proceedings of the first couple of [perinatal priorities] conferences, most of the studies were epidemiological studies, and then you'll see, I think from the early 80s onwards, the move towards more and more randomized trials and systematic reviews were being presented. And I think it was really the influence of the Oxford database of peri-natal trials which got us all thinking in that way." (Acad 3)

Collaborating closely, sharing many of the same ideas and having "members" now placed within the National Department of Health meant that this group influenced strongly the final policies and guidelines which were produced for maternal care as well as the use of evidence within these guidelines.

### Giving credibility to the policies

Respondents described how the use of research evidence was seen to lend credibility to the maternal care policies among important stakeholder groups. For example, a respondent responsible for the first draft of the 'Guidelines for Maternity Care' saw the use of evidence as not just a reflection of the values of the National Department of Health, but also of the "world that we live in" and the country. He explains:

"But I mean, in the world we live today, it is the world of evidence based practice, evidence based medicine. So one had to be careful about... cognizant of the fact that one had to use the best available evidence. Because I mean, this would be national... it would be a reflection of us as a country." (DoH 3)

He also noted how, as a consequence of his obstetrics training, evidence based medicine was "the truth I knew – that's how I was cultured" (DoH 3). In the face of a strong obstetric fraternity who were steeped in a culture of evidence based medicine, the National Department therefore had to produce guidelines that would have credibility and withstand scrutiny, as one of their officials describes:

"As I indicated...the aim of maternal and child health at national is to improve the health of women and children, and for anything to be used, especially if you include academics and everybody, for anything to be utilised, then people want to be convinced that this actually is [of] benefit and it can work. So you then need to have evidence. If you come and say that magnesium sulphate works to stop eclampsia and to further prevent people from fitting, you see people will say, 'Where do you get that from?' And you cannot say, 'I heard somebody saying it works'. You need evidence and, therefore, you have then to rely on the research that has been done. And without that information it would be very difficult to convince anybody to actually buy into what you are doing." (DoH 1)

Research evidence was seen by these respondents to have given power and credibility to these policies. The authors of the policies were keenly aware of the social context in which these policies were written. They used evidence not only because they believed that it offered the best possible treatment solution, but also because they believed that those reading the policies would be more convinced of the strength of recommendations if they were obviously supported by systematically gathered research evidence.

## Discussion

Since as early as the 1970s [37]analysts and researchers have been suggesting that the process of research utilisation is complex and politically fraught. Yet our findings could be seen as indicating a very neat assimilation of evidence into policies and clinical guidelines. Following the publication in 1995 of the results of the Collaborative Eclampsia Trial, its recommendations were adopted without change into South African maternal health care policy in order to address maternal mortality. The evidence was therefore used in an instrumental manner [7,9,38,39]. This seemingly linear assimilation of research findings into policy fits neatly within the knowledge driven model of research utilisation in which information from research is utilised because it exists [37,38]. To explain how this came about we need to look more closely at the policy process in which this research evidence was utilised. This suggests that the use of evidence was perhaps less linear than the knowledge driven model suggests and highlights the place of research networks and policy context in research utilisation.

### Getting maternal health care onto the South African policy making agenda

Drawing on Kingdon's framework [42], our findings suggest that maternal health researchers in South African acted as policy entrepreneurs in order to place issues on the government's policy agenda. Prior to the change of government in 1994, little attention was given to maternal health at a national level and there were no national policies in this area. This was despite ongoing attempts by advocates, some of whom were respondents in this study, to bring the problem of maternal health to the attention of national government. As already noted, these advocates were involved in national and international research networks for improving maternal health. They recognised the problem of poor maternal health outcomes in South Africa; they believed that there were potential solutions to this problem; and they sought an opportunity to bring government attention to this.

The political climate prior to 1994 did not, however, allow these policy entrepreneurs to gain the attention of the national government in a way that would effect meaningful policy changes. This climate changed dramatically when, in 1994, South Africa held its first democratic elections and a new national administration came into being. Following this change the National Department of Health employed a new cohort of health officials. Appointed to key positions were people with strong links to the networks of policy entrepreneurs and researchers who collectively sought a change in the approach to maternal health. These new government employees created a space in which these policy entrepreneurs could engage with the National Department of Health. As a result of their continued advocacy and the new openness in government, a decision was made to conduct a national confidential enquiry into maternal mortality. This enquiry established that maternal mortality was a problem and recommended that a national policy for maternal health be formulated. Only once the need for a national maternal health care policy was expressed did opportunities arise for drawing research evidence into that policy.

### Understanding the policy making process for maternal health in South Africa

In the context of this new drive to formulate policies, why was evidence from RCTs and systematic reviews used? Understanding the influences on policy making [40] may help us to better understand this. As alluded to above, a key event preceding the writing of the 'Guidelines to Maternity Care' was the election in 1994, which brought in a new dispensation. At an institutional level, the South African Constitution now recognised the right to health services with attention to the needs of women and children [55]. The government also prioritised women's health care in the process of reforming the National Department of Health. An important subsequent event was the first Confidential Enquiry into Maternal Deaths, the findings of which supported calls for new maternal health policies. An idea held very strongly by those charged with the responsibility for writing these policies was that these should be based on the best available scientific evidence. This was believed to be the results of systematic reviews and randomised controlled trials. Fortuitously, a randomised controlled trial showing the effectiveness of magnesium sulphate for the treatment of eclampsia was published during the period that evidence was being sought to develop policies for the management of this condition [30]. A number of key academic obstetricians in South Africa had been involved in the study, which raised its profile and gave it higher credibility within the country. Furthermore, interest groups in the new government wanted to be seen to be keeping abreast of international health care trends, including that of basing policies on rigorous scientific evidence.

Understanding the influences on policy suggests that the process of research utilisation observed in this study was more complex than first meets the eye. Although the results of the Collaborative Eclampsia Trial were published only a few years before the "Guideline for Maternity Care" was developed [30], the availability of this evidence was not sufficient to ensure its uptake automatically. Our analysis suggests that this evidence was received in a context which was open to research utilisation – when those developing policies and guidelines wanted to offer the best possible care to women. The three key categories of influence on the policymaking process – ideas, interests and institutions – were therefore in alignment, facilitating the uptake of evidence into policy making.

Our findings also highlight the interdependency of these influences on each other and the uncertain nature of the policy and guideline making process. Policies for maternal health could well have been formulated differently if the key interest groups had held a different set of values. This suggests, firstly, that networks of influence are very important and, secondly, that research utilisation can be unpredictable if dependent on the values of these networks. Our findings also confirm the importance of context to research utilisation [38]. The difficulty for those wishing to influence research utilisation is that they may not always have the power or authority to influence this context. Researchers may not be able to influence events to create windows of opportunity. However, researchers can ensure that high quality evidence is produced and is accessible to policy makers. They can also organise themselves into networks in order to enhance their influence and be willing to act as policy entrepreneurs when the opportunity arises. While the event of the South African change in government may be unique, we believe that opportunities for change in policy (policy windows) present themselves in most settings, for example when elections bring in a new government or when there is a change of health minister or senior health department officials. As Kingdon [42] suggests, policy entrepreneurs need to be attentive to when key policy influences are in alignment and when a window of opportunity opens for policy change.

### Placing the study findings in context

This study contributes to the limited body of empirical work from LMICs on the factors influencing the uptake of research into policy making [9,12]. Our study findings are consistent with those of two earlier systematic reviews [12,9] of studies in this field (see Table 1). It is also worth noting that our findings are likely to be time and context specific. For example, the policy shifts described were dependent on the change of government in 1994 and on the consequent reshaping of the policy environment.

### Limitations of the study

In exploring the role of research evidence in policy making, this study has several limitations. Firstly, it draws heavily on the accounts of respondents. A strength of this approach is that the data represents the views of those closely involved in formulating the policies studied and therefore provide valuable insights into policy processes. The interviews also provide a thick description of research utilisation processes allowing us to situate these within their wider context. This enhanced what we learnt from the experience of others. However, we recognise that such accounts are inevitably influenced by respondents' position at time of event; their position at the time of being interviewed; their relationship with the researchers; the shifts that they may have made over time between organisations; and their memory of particular events [43].

Secondly, the snowballing approach used to select respondents can yield a sample of people who share similar opinions. To avoid this, we actively sought respondents with differing views and explored negative cases [47]. Thirdly, this study did not address policy implementation, rather focusing on policy development. It is likely that resource limitations, which are often more pronounced in LMIC settings, are more important barriers to the implementation of evidence based policies than to the prior use of evidence in policy and guideline development. Further studies are required to explore the how policies for maternal health have been implemented in South Africa.

Fourth, as this is only one case study, the findings need to be generalised to other contexts and health issues with caution. As a treatment, magnesium suphate is perhaps less complex in its implementation than certain other obstetric interventions, such as the provision of emotional support during labour. However it is striking that many of our findings reflect those of the systematic reviews discussed earlier. Furthermore, this is not the only example of evidence being applied to treatment policy in South Africa. In a similar example of linear research utilisation, evidence for the effectiveness of artemisinin-based combination therapy in the treatment of uncomplicated *Plasmodium falciparum *malaria was used to change malaria treatment policy in the KwaZulu-Natal province [56]. Although the case study approach, like many qualitative methods, has been critiqued for having limited generalisability [57], we would argue that the approach allows theoretical generalisability [52] and also provides insights into the ways in which knowledge informs policy making in 'real life' contexts [58]. The transferability of the findings reported here will also be enhanced when considered alongside those from the other five cases, which contribute to the main study [36].

## Conclusion

This study provides an example of the direct use of research evidence in the development of maternal health policies and guidelines. However, we would also argue that the use of research findings in this case had symbolic value, indicating to the potential users of the policies and to other actors that South Africa was part of the broader international movement to base clinical policies and practice on high quality research evidence. In this and a number of other ways, this study illustrates how policies are shaped by the national and international contexts in which they are developed. The impact of context is further highlighted by the finding that the uptake of RCT evidence on the effectiveness of magnesium sulphate in treating eclampsia into clinical policies was facilitated by the opening of a window of opportunity, following the change of government in South Africa in 1994. This window allowed a network of researchers to place maternal health on the agenda of policy makers. This study therefore also indicates the ways in which researchers may not only influence the content of policies and guidelines but also, through acting as policy entrepreneurs, shape policy agendas [42]. We would not, however, wish to overstate the role of research evidence in driving policy development for maternal health in South Africa. As we have discussed, such evidence was just one of a constellation of factors influencing this process (see Table 1) and it was certainly serendipitous that an important international RCT, in which a number of key South African researchers had participated, was published shortly before policies for the management of eclampsia were developed. Nonetheless, the close links between the research and policy arenas were central to the emergence of evidence based policies for maternal health in South Africa.

## Abbreviations

MgS04: magnesium sulphate; LMIC: low and middle income countries; NCCEMD: National Committee for Confidential Enquiry into Maternal Deaths; RCT: randomised controlled trial; Acad: Academic researcher and clinician; DoH: Former or current national Department of Health official; (Respondents are differentiated by number in the text, e.g. Acad 1, DoH 3.)

## Competing interests

The authors declare that they have no competing interests.

## Authors' contributions

KD conducted the research and wrote the paper supervised by SL who was the principal investigator for the study in South Africa. They were actively supported in the process by the Practihc Policy Group.
